# First Case Report of Sinusitis with* Lophomonas blattarum* from Iran

**DOI:** 10.1155/2016/2614187

**Published:** 2016-02-04

**Authors:** Fariba Berenji, Mahmoud Parian, Abdolmajid Fata, Mahdi Bakhshaee, Fereshte Fattahi

**Affiliations:** ^1^Department of Parasitology and Mycology, Medical School, Mashhad University of Medical Sciences, Mashhad 91779-48564, Iran; ^2^Ear, Nose and Throat Department, Medical School, Mashhad University of Medical Sciences, Mashhad, Iran; ^3^Mashhad University of Medical Sciences, Mashhad, Iran

## Abstract

*Introduction*.* Lophomonas blattarum* is a rare cause of bronchopulmonary and sinus infection. This paper presents a rare case of* Lophomonas* sinusitis.* Case Presentation*. The patient was a 31-year-old woman who was admitted because of a history of upper respiratory infection and sinusitis. Direct microscopic examination of the sputum and nasal discharge showed large numbers of living* Lophomonas blattarum* with irregular movement of flagella. The patient was successfully treated by* Metronidazole* 750 mg t.i.d. for 30 days.* Conclusions*. This is the first case report of* Lophomonas blattarum* sinusitis from Iran.

## 1. Introduction

Improving society's overall hygiene level contributes to prevention and control of parasitic infections. However, other factors including insects' infestation may complicate control and eradication of parasitic infection.

Hypermastigida, together with Trichomonadida, is an order of multi flagellated protozoa belonging to the phylum Parabasalia. This order consists of two suborders: Lophomonadidae and Trichonymphina. Lophomonadidae can be parasitic or symbiotic in the digestive system of termites, cockroaches, and wood roaches [1].* Lophomonas blattarum* was first described by S. Stein in 1860, from the gut of the cockroach* Blatta orientalis* [2]. This protozoan is round to oval shape and 20–60 *μ*m in diameter with an apical tuft of numerous flagella.* Lophomonas blattarum* is a rare cause of bronchopulmonary infection and respiratory symptoms [2].* Lophomonas blattarum* can occasionally infect humans. Only a few articles reported* L. blattarum* and other flagellated protozoa causing bronchopulmonary infections in humans [3–5]. These protozoa more commonly infect immunocompromised individuals [6]. Most reported cases are from China and few from Turkey [7, 8]. This report is the first case of* L. blattarum* human infection in Iran.

## 2. Case Presentation

The patient was a 31-year-old woman who was admitted on September 13, 2014, because of a history of upper respiratory infection and sinusitis developed rapidly with progressive headache, fever, dizziness, pain of left ear, and sneezing. The nasal discharge and sputum were occasionally green and sticky. Preadmission antibiotic therapy with Cephalexin was not effective. Ear, Nose, and Throat examination revealed congestion and erythema of nose and throat but normal ears. The patient was a lab technician and made direct smears from her nasal discharge and sputum. Surprisingly she observed moving flagellated protozoa.

**Figure 1:**
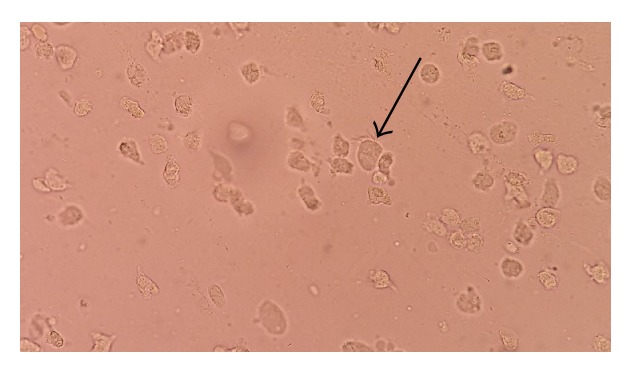
*Lophomonas blattarum* in direct smear of nasal discharge (40x).

After referring to our parasitology lab, direct microscopic examinations of the sputum and nasal discharge showed large numbers of living* L. blattarum* with irregular movement of flagella (Figure 1).

Laboratory examination demonstrated a white blood cell count of 8.7 × 10^3^ per liter 7% eosinophils, ESR of 27, and positive C-Reactive Protein. Microbial culture for detection anaerobics was negative.

Computed tomography (CT) scan of sinuses demonstrated bilateral maxillary mucosal thickening accompanying osteomeatal complex (OMC) obstruction. Mild ethmoidal involvement especially in left side without bone destruction was considered. Other paranasal sinuses were normal in point of pneumatization without any involvement.

There were no respiratory sign and symptoms. Chest X-ray was normal.

The patient was treated with* Metronidazole* 500 mg b.i.d. for 15 days. Her symptoms were relieved after treatment; however, a few protozoa were found in microscopic direct smears of nasal discharge. Additional treatment with* Metronidazole* 750 mg t.i.d for 15 days cleared the nasal smear.

## 3. Discussion

In recent years there has been an increase of reports covering* Lophomonas blattarum* bronchopulmonary infection, most notably 60 cases from South of China [2]. There were additional cases from other countries including Spain and Turkey [9].* Lophomonas* sinusitis, on the other hand, has been rarely reported. This case of* Lophomonas* sinus infection is the first reported in Iran. Most cases of Lophomonas infection reported in immunocompromised patients [9], but this present case is an immunocompetent patient. Since the real mechanism of transmission has not been clearly described, it is important for clinicians to consider* Lophomonas blattarum* in their differential diagnosis. Additionally, since similar flagellate has been reported in house dust mite intestine there may be a relationship between respiratory allergy and* Lophomonas blattarum* or other flagellate [3]. Therefore, in patients with chronic allergy with no response to treatment, Lophomonas infection and* Metronidazole* treatment may be considered.
